# Wheelchair tiedown and occupant restraint practices in paratransit vehicles

**DOI:** 10.1371/journal.pone.0186829

**Published:** 2018-01-05

**Authors:** Karen Frost, Gina Bertocci, Craig Smalley

**Affiliations:** Department of Bioengineering, J.B. Speed School of Engineering, University of Louisville, Louisville, Kentucky, United States of America; University of Illinois at Urbana-Champaign, UNITED STATES

## Abstract

The purpose of this study was to characterize wheelchair tiedown and occupant restraint system (WTORS) usage in paratransit vehicles based on observations of wheelchair and scooter (wheeled mobility devices, collectively, “WhMD”) passenger trips. A retrospective review of on-board video monitoring recordings of WhMD trips was conducted. Four hundred seventy-five video recordings were collected for review and analysis. The use of all four tiedowns to secure the WhMD was observed more frequently for power WhMDs (82%) and manual WhMDs (80%) compared to scooters (39%), and this difference was significant (p< 0.01). Nonuse or misuse of the occupant restraint system occurred during 88% of WhMD trips, and was most frequently due to vehicle operator neglect in applying the shoulder belt. Despite the absence of incidents or injuries in this study, misuse and nonuse of WTORS potentially place WhMD seated passengers at higher risk of injury during transit. These findings support the need for improved vehicle operator training and passenger education on the proper use of WTORS and development of WTORS with improved usability and/or alternative technologies that can be automated or used independently.

## Introduction

Wheeled mobility device (WhMD) users who do not have access to private accessible transportation or are unable to use public transit buses despite accessibility features are reliant on paratransit services. Paratransit vehicles commonly used to transport WhMD-seated passengers include full size modified vans and mini-buses equipped with lifts. These vehicles are considered high-*g* environments because they have a relatively low mass[1] compared to larger transit buses, resulting in increased crash severity and a greater potential for injury during a crash or emergency maneuvering. The deceleration pulse experienced by paratransit vehicles during a frontal impact can be three times as high as those associated with a larger transit bus exposed to the same frontal impact[2], requiring the use of wheelchair tiedowns to secure the wheelchair, and occupant restraints (lap and shoulder belts) to ensure occupant protection for WhMD users and nearby passengers.

**Fig 1:**
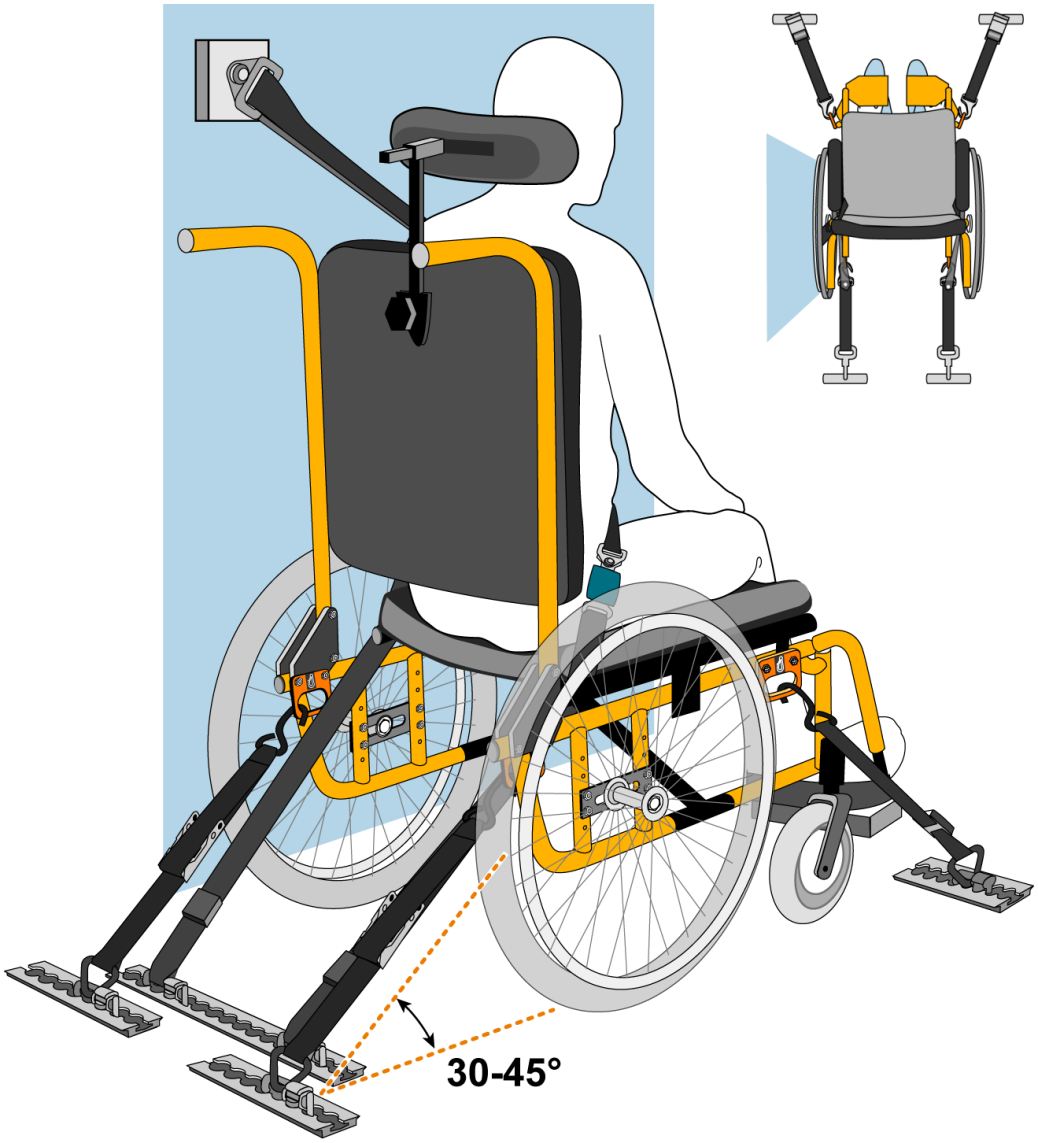
Wheelchair tiedown and occupant restraint system (WTORS). WTORS consisting of two tiedowns securing the front of the wheelchair, two tiedowns securing the rear of the wheelchair, and including both a lap and shoulder belt for occupant restraint (color figure available online).

Four-point, strap-type wheelchair tiedown and occupant restraint systems (WTORS) are the primary means of securing WhMDs and restraining occupants in public transit vehicles in accordance with the accessibility requirements of the American with Disabilities Act[3]. As detailed previously[4], WTORS consist of four wheelchair tiedown (WT) straps and an occupant restraint system (ORS) comprised of a shoulder and lap belt assembly used to restrain the passenger’s torso and pelvis. Proper use of the WTORS is shown in Fig 1 and requires using all four tiedown straps to secure the WhMD to the vehicle floor and tensioning the straps to achieve a 30–45 degree angle[5].

Despite the presence of WTORS, the injury incidence rate for paratransit WhMD passengers is estimated at 10 injuries per 100,000 trips, compared to an incidence rate of 2–6 injuries per 100,000 trips for all passengers[6]. Wretstrand et al. found that 12% of WhMD passengers reported being involved in an incident while using paratransit services in Sweden. The most common cause of incidents was vehicle braking or acceleration during non-crash events; approximately two-thirds of incidents occurred during normal driving or sudden maneuvering[7]. Injuries varied from minor to severe, but the majority of passengers received medical treatment. Interestingly, 94% of passengers stated their WhMD was always secured, and 78% reported always using occupant restraints. Despite the high reported usage rate of WTORS, adverse incidents and injuries remained high.

Our previous investigation of WTORS usage in public transit buses, and the research of others suggest that WTORS may not be implemented or used properly in the field, potentially placing wheelchair users at increased risk of injury[8–12]. In this study, we reviewed video recordings from on-board vehicle cameras for the purpose of describing *in situ* use of WTORS during paratransit travel.

## Methods

Approval to conduct this study was obtained from the University of Louisville Institutional Review Board (IRB No. 14.0274) through the Expedited Review Procedure in accordance with 45 CFR 46.110(b), Category 7. Additionally, a waiver of informed consent was granted according to 45 CFR 46.116 (D).

### Transit agency and ridership

The study was conducted in a metropolitan transit agency located in the southeastern region of the United States. The transit agency owns 85 paratransit vehicles and provides door-to-door service. Weekly WhMD boardings are estimated at 600–700; annual WhMD trips average 33,800. All wheelchair accessible paratransit vehicles are equipped with WTORS, and signs are posted on each vehicle notifying passengers that activities within and outside the vehicle are being monitored and recorded for public safety purposes. The transit agency randomly assigns operators to vehicles on a daily basis, thus all operators had an equal likelihood of being assigned either vehicle during the course of this study.

### Video recordings

**Fig 2:**
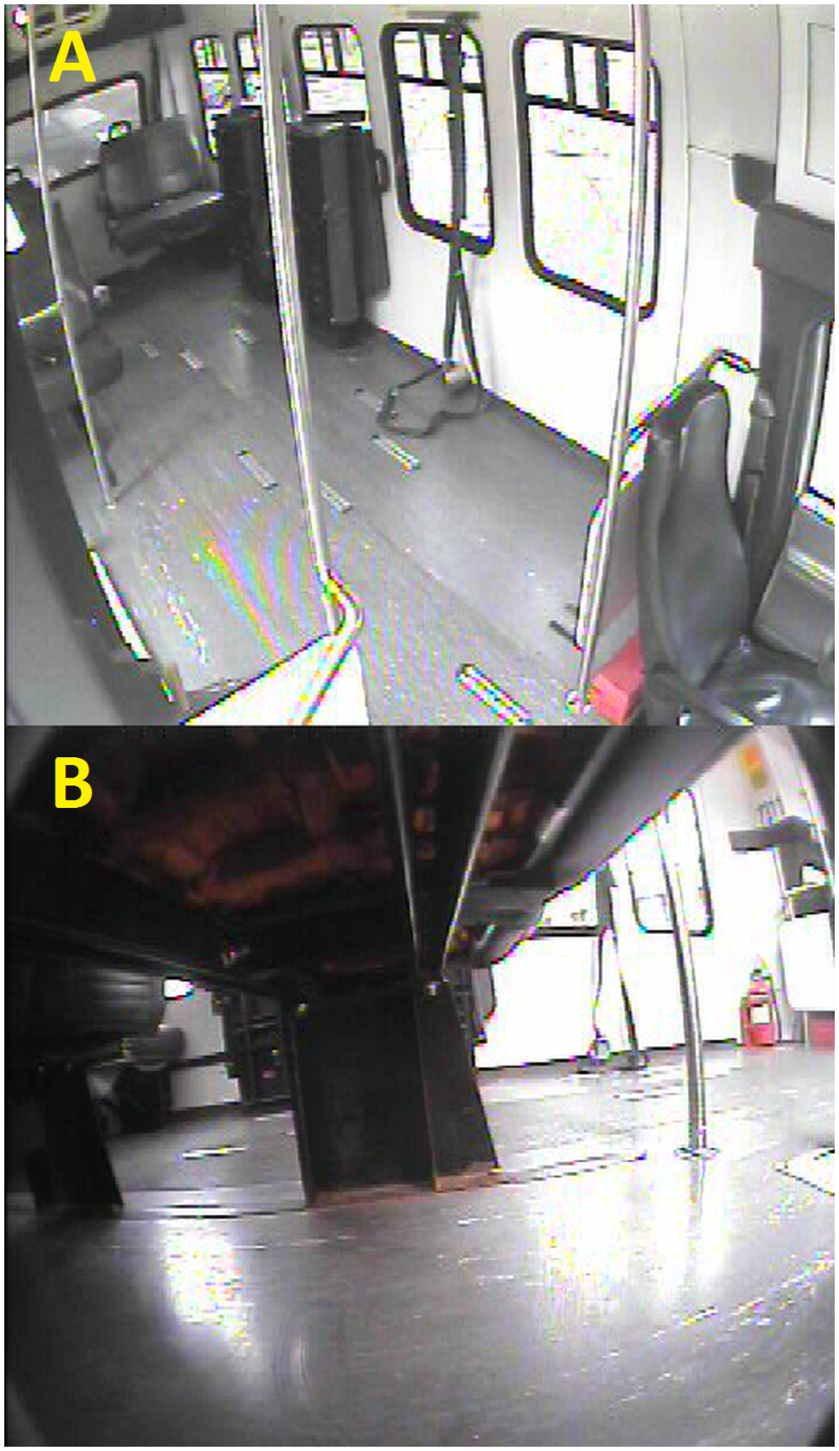
Sample images of WhMD securement station from video surveillance system (AngelTrax^®^). (A) Overhead view, (B) floor level view.

Video recordings of WhMD activities on two paratransit vehicles were retrospectively reviewed by the research team. The first vehicle was a 2011 General Motors, Chevrolet^®^ G3500 paratransit vehicle equipped with a rear mount Braun Millennium^®^ 2 lift. The second vehicle was a 2006 Ford Econoline^®^ E350 paratransit vehicle equipped with a side mount Ricon^®^ S5005 lift. Each vehicle was equipped with a dashboard-mounted camera (“dashcam”) providing a view of the vehicle interior, however, additional cameras were needed to fully observe the use of WTORS. Thus each vehicle was additionally equipped with a 3-camera AngelTrax^®^ MiniMicro video surveillance system consisting of high resolution, multi-purpose interior cameras, each having a 90-degree adjustable mount, and equipped with infrared capability to record high quality video in both day and night conditions. Cameras were located to capture views of vehicle operators applying the WTORS, and lift boarding and alighting activities (Fig 2). Additionally, the 2006 Ford Econoline^®^ van was equipped with a fourth camera that provided a floor level view of the wheelchair tiedown system. Video image resolution, frame rate and storage are described elsewhere[4].

### Data collection and analysis

Data collection occurred over a 12-month period to allow for collection during all four seasons. Video records were downloaded on a bi-weekly basis to a laptop computer in the field using a USB cable, and viewed and abstracted using AngelTrax’s FlexPlay^™^ software. Video review was performed by two of the authors (CS and KF) using methods previously described to insure consistency between reviewers[4]. WTORS usage and WhMD-related data were recorded as categorical or continuous data and abstracted into a customized database (FileMaker Pro 12 Ver. 3). WhMD-related data included WhMD type, number of items (backpacks/bags) attached to the WhMD, and any additional assistive technology used or carried by the WhMD passenger that may have encumbered use of the WTORS.

WTORS usage was subdivided into two categories: WhMD tiedown usage or occupant restraint usage. WhMD tiedown data was recorded based on whether or not WhMD tiedowns were used, along with the number and location of the applied tiedowns (as applicable). Tiedown usage was further subcategorized as follows: i) “complete use” was defined as the use of all four tiedowns, ii) “misuse” was defined as the use of between one to three tiedowns, incorrect placement of the tiedown on the WhMD (or of the tiedown anchor point on the vehicle floor), overtly incorrect tiedown strap angle, and/or excessive slack in tiedown straps, and iii) “nonuse” was defined as situations in which zero tiedowns were applied to the WhMD. The same terms were used to subcategorize occupant restraint usage, where i) “complete use” was defined as the use of both the shoulder and lap belt, ii) “misuse” was defined as neglecting to use either the lap or shoulder belt, or incorrectly routing one or both belts, and iii) “nonuse” was defined as situations in which neither the shoulder nor lap belt were used to restrain the occupant. Also recorded was the individual who applied the WTORS (vehicle operator, caregiver/assistant, other passenger).

Descriptive statistics were used to present all data. Post-hoc independent samples chi-square analysis was performed to examine WTORS usage based on WhMD type and vehicle. All statistical analysis was conducted using SPSS Statistics, Ver 22 (IBM^®^).

## Results

Video records of 475 WhMD trips were collected over a 12-month period of time covering each weather season. As previously stated, the transit agency randomly assigns operators to vehicles each day.

Slightly more power WhMDs (n = 234, 49.3%) were observed than manual WhMDs (n = 222, 46.7%). Few scooters were observed (n = 19, 4.0%). Most passengers remained seated in their WhMD with 33 (7%) transferring to a vehicle seat. Backpacks or bags were attached to 64.2% of WhMDs (n = 305), with 8.8% (n = 42) carrying multiple backpacks/bags; the majority attached to the rear of the WhMD (n = 281, 81.2%). Seventy-six WhMDs (16.0%) had elevated footrests, and 6 (1.3%) had reclined seats. Footrests were considered elevated if the angle between the footplate and vehicle floor appeared to exceed 20 degrees. Seatbacks were considered reclined if the angle between the seatback and wheelchair frame appeared to exceed 110 degrees.

### Tiedown usage

The vehicle operator applied the WTORS in nearly all cases (n = 470, 99%); in 4 cases (>1%) an assistant of the passenger or other person applied the WTORS. In one trip, zero WTORS were applied.

**Fig 3:**
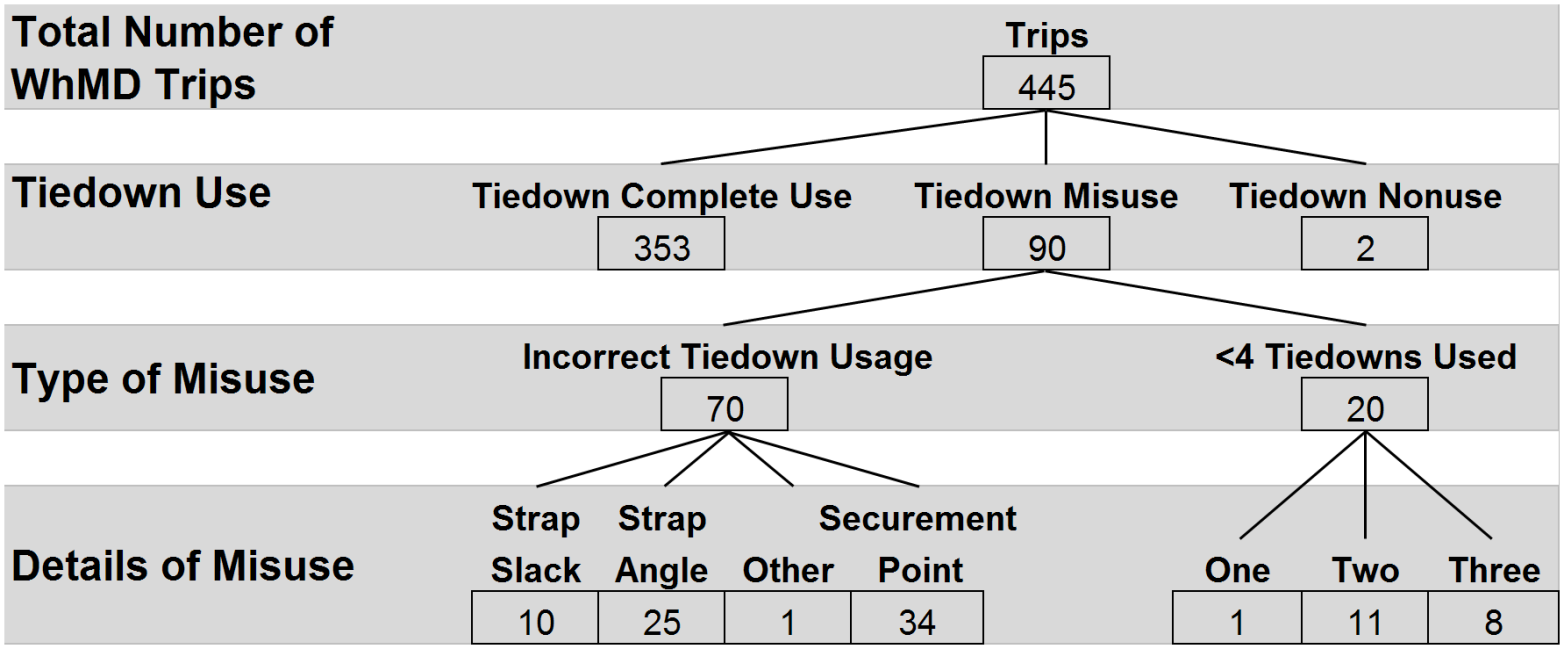
Summary of wheelchair tiedown usage.

The use of all four tiedowns was observed during the majority of trips (n = 353, 75%). Tiedown nonuse was observed in only two trips. Misuse was observed during 90 trips (19%) and is detailed in Fig 3. Among WhMDs having elevated footrests (n = 76), incorrect placement of front tiedowns on the casters or footrest bar was observed in 7% of cases. Tiedown usage could not be classified for 30 trips (6%) in which obstructions prevented observation of tiedown usage and/or securement locations on the WhMD.

### Tiedown usage by WhMD

**Fig 4:**
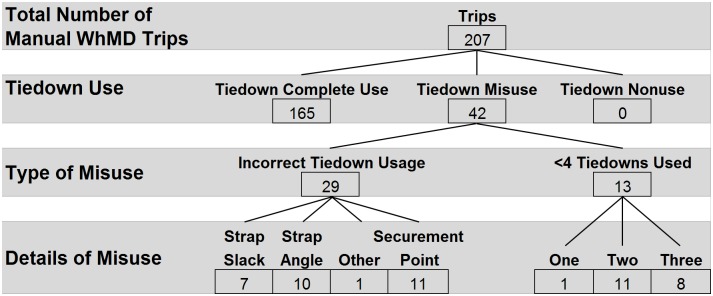
Wheelchair tiedown usage for manual WhMDs.

Details of tiedown usage for 207 manual WhMD trips revealed complete use of tiedowns during most trips (n = 165, 80%) and zero instances of nonuse (Fig 4). Misuse was observed during 42 trips involving manual WhMDs; incorrect placement of the tiedown on the WhMD occurred during 11 trips (26%), 10 instances involved attachment of a tiedown to a caster, one instance involved attachment to a footrest, and one instance involved improperly looping a tiedown through the WhMD frame. An overtly incorrect strap angle was observed in 10 trips (24%) and excessive slack in one or more straps, evidenced by the tiedown strap sagging or laying on the vehicle floor, was observed in 7 trips (17%). Nearly a third of observations involved the use of less than four tiedowns (n = 13, 31%).

**Fig 5:**
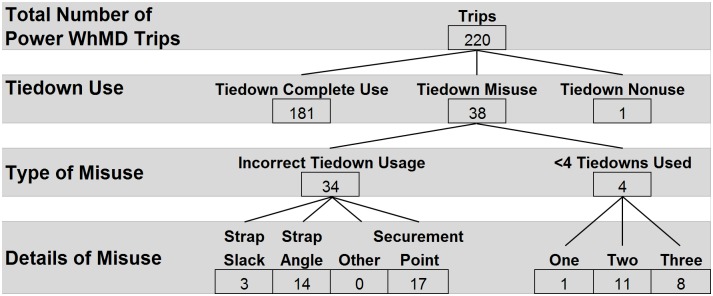
Wheelchair tiedown usage for power WhMDs.

Tiedown usage for power WhMDs (n = 220) followed a pattern similar to that of manual WhMDs (Fig 5). Complete use of tiedowns was observed in the majority of trips (n = 181, 82%) and nonuse was observed once (0.1%). Among 38 misuse cases, incorrect securement location was most common (n = 17, 45%). Instead of being attached to the WhMD frame, tiedowns were attached to armrests (n = 8, 21%), casters (n = 6, 16%), seat posts (n = 2, 5%), and in one instance, a footrest (n = 1, 3%). Overtly incorrect strap angle was observed in 14 trips (37%), and excessive slack in tiedown straps was observed in 3 trips (8%).

**Fig 6:**
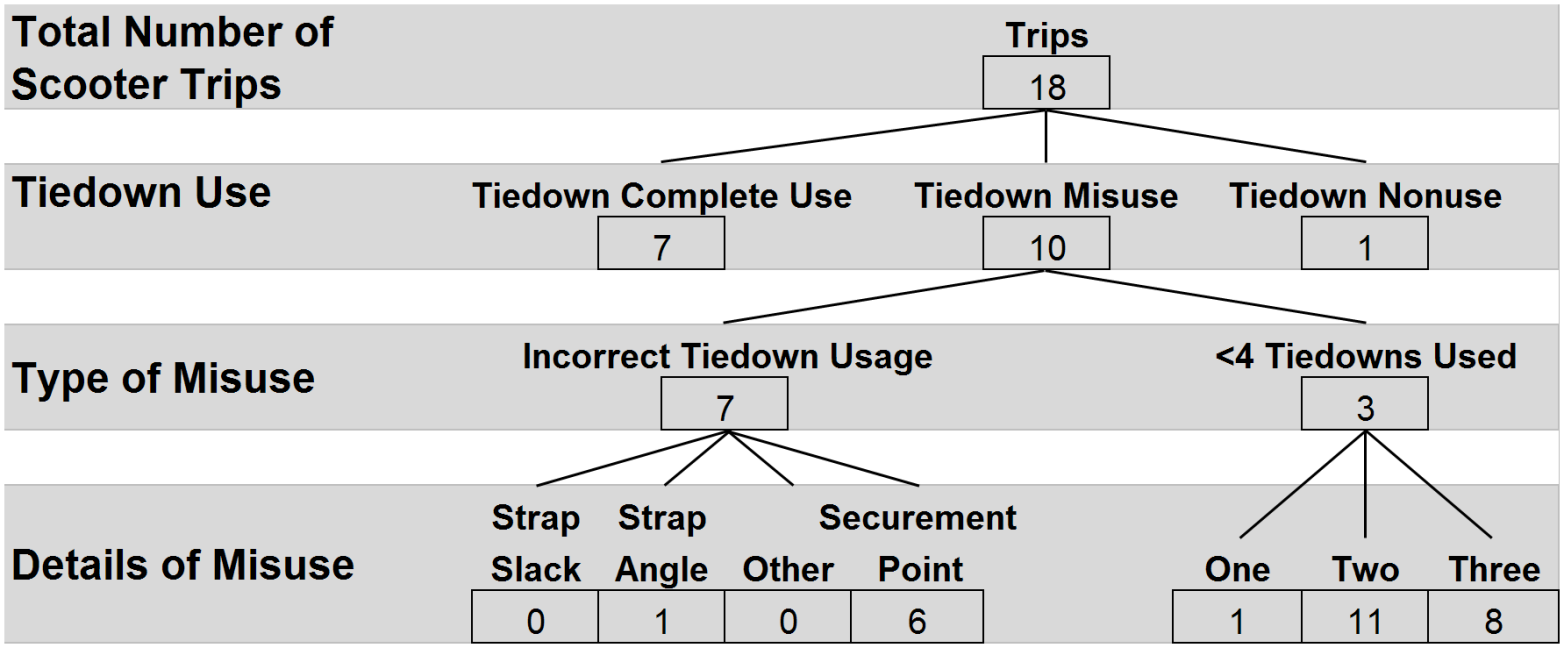
Wheelchair tiedown usage for scooters.

Tiedown usage was observed during all but one scooter trip (Fig 6). Complete use of tiedowns occurred during 7 trips (39%). However, the majority of trips involved misuse of tiedowns (n = 10, 56%). Of the 10 misuse cases, incorrect placement of the tiedown on the scooter tiller occurred most frequently (n = 6, 60%), followed by use of less than four tiedowns (30%). One instance of tiedown nonuse was observed (6%).

### Post-hoc analysis—Tiedown usage

Post-hoc chi-square analysis was conducted to examine tiedown usage according to vehicle, WhMD type, and whether or not the passenger remained seated in their wheelchair during transit. Results indicated vehicle operators were less likely to use all four tiedowns to secure scooters compared to manual or power WhMDs (χ^2^(2) = 19.13, p<0.01), and twice as likely to use all four tiedowns when passengers *remained seated in their wheelchair* during transit compared to practices used to secure an unoccupied WhMD (χ^2^(2) = 104.594, p<0.01). There was no difference in tiedown practices based on vehicle (complete tiedown use versus misuse;χ^2^(1) = 0.196, p = 0.66).

### ORS usage

**Fig 7:**
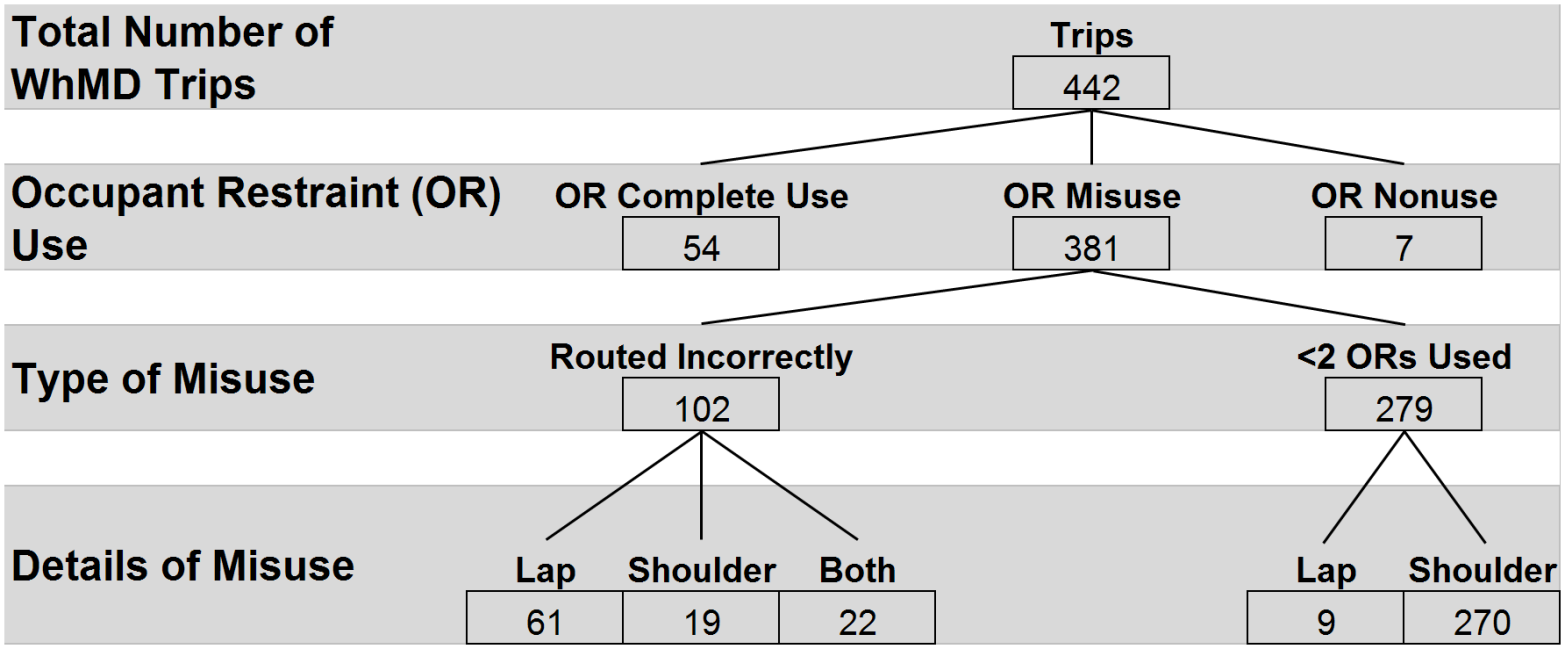
Summary of occupant restraint usage.

Analysis of ORS usage excludes 33 trips in which the passenger transferred from their WhMD to a vehicle seat (Fig 7). Overall, ORS was most frequently misused (n = 381, 86%), either by incorrectly routing one or both belts, or neglecting to apply the shoulder belt. The majority of incorrectly routed lap belts were placed on top of the armrests, followed by those placed in front of the armrest or vertical tubing/supports. Shoulder belts were frequently incorrectly routed *between* the occupant and the seatback. Complete use of the ORS was observed in just 54 trips (12%); nonuse was observed in 7 trips (2%).

### ORS usage by WhMD

**Fig 8:**
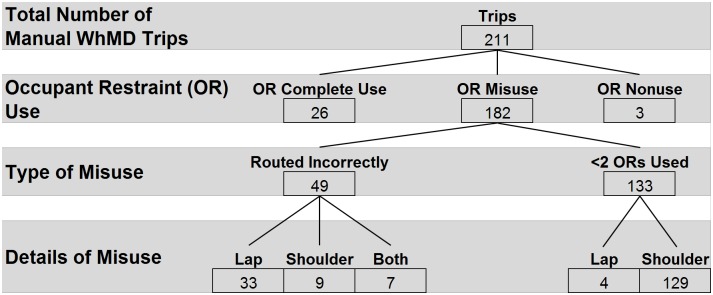


**Fig 9:**
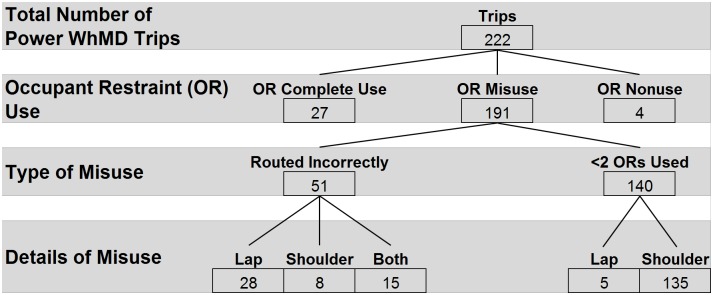


**Fig 10:**
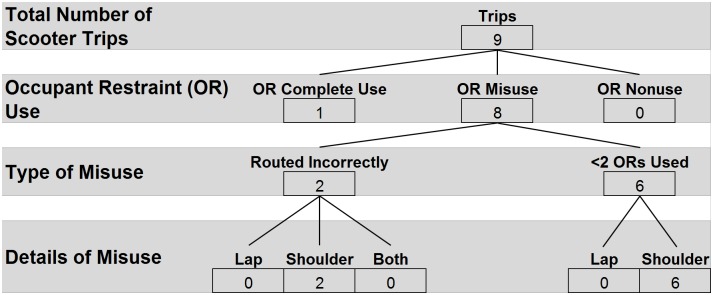


ORS usage followed a similar pattern during both manual and power WhMD trips, (Figs 8 and 9). Eighty-six percent of observations revealed the ORS was most frequently misused as a result of vehicle operators neglecting to apply the shoulder belt. Incorrect routing of the lap and shoulder belts, and nonuse of occupant restraints were observed with similar frequency (23% and 5%, respectively). During scooter trips, complete use of the ORS was observed once (11%), and misuse was observed in the remaining 8 trips (89%), typically consisting of the vehicle operator neglecting to use the shoulder belt (Fig 10).

### Post-hoc analysis—ORS usage

Post-hoc Chi-square analysis to assess the relationship between ORS usage and WhMD type revealed no significant relationship between type of WhMD and correct use versus misuse of lap (χ^2^(1) = 0.000, p>.05) or shoulder (χ^2^(2) = 0.105, p>.05) belts. Scooters were excluded from this analysis as the distribution of misuse among scooters did not meet the assumptions for the Chi-square statistic. Nor was there a difference between vehicles in the application (complete use versus misuse) of lap or shoulder belts (χ^2^(1) = 0.006, p = 0.94 and χ^2^(1) = 3.578, p = 0.06, respectively).

## Discussion

To our knowledge, this is the first observational study to report on WTORS practices in a paratransit environment. Our objective was to characterize WTORS usage to provide an understanding of real world practices so that transit policies, procedures and vehicle operator training can be optimized for improved WhMD user safety when traveling in high-*g* environments such as paratransit vehicles. Best practice dictates the use of 4 tiedown straps to secure the WhMD, and the use of both lap and shoulder belts to restrain the occupant.

We were alarmed to find that the majority of WhMD passengers were not afforded the benefit of occupant restraints despite transit agency policy mandating their use. Video observation revealed that vehicle operators failed to apply one or both occupant restraint belts during 65% of trips (Fig 7). Failure to apply the shoulder belt accounted for 98% of these cases, and consisted of the vehicle operator allowing the shoulder belt to hang loose behind passenger’s WhMD. This finding is comparable to our previous report of no observations of ORS use in fixed route public transit buses[11], however this comparison is limited as transit agency policy does not mandate the use of ORS in public transit buses.

In general, it is recommended that WhMD passengers transfer to an OEM floor-mounted vehicle seat provided they have adequate postural stability and the opportunity to utilize an ORS. In this study, only 8% of WhMD passengers transferred to vehicle seats, resulting in a majority of passengers who were reliant on the vehicle operator to properly apply the WTORS for their safety. WhMD users are a particularly vulnerable group of paratransit passengers. They are more likely to be elderly, frail and/or have less physical function, making them less able to transfer to a vehicle seat or to stabilize themselves while seated in their WhMD. Despite the absence of injuries in our study, our findings suggest a high potential for adverse incidents given poor compliance with best WTORS practices.

Although tiedown use to secure WhMDs exceeded that of ORS, 1 in 5 WhMDs were not completely secured using all four tiedowns, increasing the potential for WhMD movement or tipping during vehicle maneuvers or when the vehicle is in motion. In Wrestrand’s study, 89% of injury incidents that occurred when the paratransit vehicle was moving occurred during routine driving manuevers[6]. He also reported that the injury incidence rate for paratransit WhMD passengers is almost twice that of ambulatory and WhMD passengers combined, underscoring the need for WTORS usage. Salipur et al. (2012) reported WhMD instability during 20% of observed WhMD trips in public transit buses, a percentage that is likely lower than would be expected in paratransit vehicles given the low-*g* environment of transit buses[13]. In our study we observed several instances in which the vehicle operator stopped driving and returned to the securement station to re-position and re-secure the WhMD. In one trip, a tiedown detached from a WhMD during transit, and in another, an unoccupied manual wheelchair “secured” using only a shoulder strap tipped over. None of these instances resulted in an adverse consequence, but they raise concern as to WTOR strap nonuse and lack of proper strap tensioning.

Our methodological approach using video records proved useful in characterizing types of misuse and nonuse. It also adds to the growing body of evidence highlighting existing challenges with the use of WTORS. Alternative strategies, such as passive or automated rear-facing WhMD retainment stations (e.g. Quantum^®^, Q’Straint) are beginning to be used in public transit buses in the US and are commonly used by Canadian and European transit agencies. However, this approach is not appropriate in a paratransit environment given the potential for high-*g* crashes.

Despite sampling from a single transit agency, our findings may be of benefit throughout the transportation industry to improve vehicle operator training and transit policies and procedures for WTORS use. A better understanding of WTORS misuse and nonuse may inform transit agency supervisors whose job is to investigate incidents and perform “ride alongs” to observe and improve vehicle operator practices. Additionally, knowledge of repeated types of misuse can potentially direct WTORS manufacturers to modifications that may be warranted in subsequent design revisions. Moreover, understanding WTORS misuse and nonuse can potentially guide vehicle manufacturers in improving the design of WhMD securement spaces, and provide rationale for policy makers seeking to improve WhMD transportation safety.

Our findings are not without limitations. As mentioned, our observations were limited to that of a single transit agency. Our assessments of video footage only determined frequency and type of WTORS use. We were unable to determine WTORS tension or angles, and in some cases, the exact location of a tiedown on the WhMD. Adding a qualitative component to this study may have allowed us to gain insights specific to cases of misuse and nonuse from the perspective of vehicle operators and WhMD passengers.

## Conclusions

Observation of vehicle operator practices at a single transit agency was useful in characterizing WTORS misuse and nonuse. These practices affect a majority of WhMD passengers who are reliant on paratransit services. Our primary finding of nonuse or misuse of the occupant restraint system during 88% of WhMD trips, combined with incomplete use of tiedowns to secure 1 in 5 WhMDs supports the need for improved vehicle operator training and passenger education on the proper use of WTORS. Our findings also contribute to the growing body of evidence indicating these systems are not being properly utilized in the field, and the need for development and implementation of WTORS with improved usability and/or alternative technologies that can be automated or used independently.

## Supporting information

S1 Fig Wheelchair tiedown and occupant restraint system (WTORS). Reprinted from Ride Safe (http://wc-transportation-safety.umtri.umich.edu/ridesafe-brochure) under a CC-BY license, with permission from the Regents of the University of Michigan, original copyright 2015.
(PDF)

## Abbreviations

ADA: Americans with Disabilities Act
OEM: Original equipment manufacturer
ORS: Occupant restraint system
WhMD: Wheeled mobility device
WTORS: Wheelchair tiedown and occupant restraint system
