# Supplementary material for: Wheelchair tiedown and occupant restraint practices in paratransit vehicles
Source: PLoS One. 2018 Jan 5;13(1):e0186829. doi: 10.1371/journal.pone.0186829 (PMC5755728; doi:10.1371/journal.pone.0186829)
Supplement: S1 Fig — Reprinted from Ride Safe (http://wc-transportation-safety.umtri.umich.edu/ridesafe-brochure) under a CC-BY license, with permission from the Regents of the University of Michigan, original copyright 2015. (PDF) [file pone.0186829.s001.pdf]

From: **Miriam Manary** mmanary@umich.edu
Subject: Permission to use RideSafe graphic.
Date: August 26, 2017 at 8:11 AM
To: karen.bertocci@louisville.edu

MM

Karen,

Thanks for requesting this permission. I am able to grant you permission to publish the RideSafe graphic shown in Figure 1 of your manuscript under the Creative Commons Attribution License (CCAL) CC BY 4.0 provided you include appropriate attribution. This figure is from the Ride Safe brochure (http://wc-transportation-safety.umtri.umich.edu/ridesafe-brochure) published by the University of Michigan Transportation Research Institute (2015). The original copyright is held by the Regents of the University of Michigan, 2000.

- Miriam
